# Factors driving FIFA world cup 2022 viewership ratings in mainland China: marketing outlooks for FIFA world cup 2026

**DOI:** 10.3389/fspor.2023.1282898

**Published:** 2024-01-08

**Authors:** Zongqing Wang, Lide Su, Agudamu, Tao Gong, Te Bu, Yang Zhang

**Affiliations:** ^1^ College of Physical Education, Hunan Normal University, Changsha, China; ^2^ School of Humanities, Inner Mongolia University of Technology, Hohhot, China; ^3^ Graduate School of Social Welfare, Sungkyunkwan University, Seoul, Republic of Korea; ^4^ Institute of Sports and Health Industry, HEHA CAT Fitness, Changsha, China; ^5^ Independent Researcher, Windermere, FL, United States

**Keywords:** mega-events, football, GAMLSS, globalization, TV audience

## Abstract

**Introduction:**

The FIFA World Cup is not only the most lucrative athletic event globally, but it also functions as a platform for promoting peace owing to FIFA's new vision. Nevertheless, the determinants of TV viewership ratings, especially in the Chinese market, which is a critical revenue stream for FIFA TV broadcasting, are still unsolved. Using a distributional regression, this study aimed to quantify the dynamics of viewership ratings for the FIFA World Cup 2022 in mainland China.

**Methods:**

The CCTV viewership ratings were modeled using 12 covariables related to Chinese TV consumer behavior. Given the data structure, a Logit Normal regression model was chosen to fit the location and dispersion parameters of viewership ratings to explanatory variables.

**Results:**

In the fitted heteroscedastic model, the viewership ratings dynamics in mainland China were driven by the match kick-off time: $\hat{\mu }=\mathrm{logistic}[-4.874+0.043\times \mathrm{match}\;\mathrm{kick}\text{-}\mathrm{off}\;\mathrm{time}]$. In addition, the model captures the factors that influence the variations in viewership ratings: $\hat{\sigma }=\exp[-14.26\text{–}1.346$ (if, FIFA World Cup champion = “Yes”) + 0.004 × FIFA world ranking]. Thus, it shows that the FIFA World Cup champions tamp down such variations, leading to a more stable viewing behavior among Chinese consumers.

**Conclusions:**

Time- and team-sensitive strategies are proposed to aid in crafting uncertainty-suppressing business decisions for the FIFA World Cup 2026. Ultimately, in the more insecure 2020s, a broader live coverage of the FIFA World Cup would be invaluable for promoting peace.

## 1 Introduction

Modern sports are characterized by their freedom, equality, fair competition, the pursuit of excellence, and sportsmanship. These spirits have a universally recognized value and identity, which aligns with corporate values and social responsibility. Nowadays, sports, business, and globalization are intertwined (1), as businesses enhance their global corporate image by underwriting major sporting events and as sports competitions have transformed into international business competitions. Beyond the Olympics, the FIFA World Cup is the most prestigious global sporting event with extraordinary commercial value (2), which plays an imperative role in facilitating entry into foreign markets and enhancing the global reach of corporations (3). Marketing at the FIFA World Cup encompasses different forms, such as sponsoring finalist teams and securing exclusive commercial rights by recruiting soccer stars. Among those, advertising during live broadcast matches is the most popular form of marketing. According to FIFA's 2018 financial records (4), the sale of TV broadcasting rights accounted for 55% of the total revenue, underscoring the crucial role of TV viewership in advertising for the FIFA World Cup. In a different light, the competition for regional TV broadcasting rights reaches exorbitant levels of cost. China Central Television (CCTV) obtained exclusive media rights for the FIFA World Cup 2022, although the exact amount they paid for these rights has not been made public. CCTV then entered into a sub-licensing agreement with Migu Culture Technology and Douyin Group, reportedly for a sum of 1 billion Chinese yuan. In essence, enterprises that bid on advertising rights during live broadcasting strive to maximize their brand visibility, and in this context, viewership ratings are the most important metrics for gauging the success of a strategic business decision.

Without a comprehensive analysis of the factors driving viewership ratings, businesses cannot make a high-yield investment decision. Failure to do so could result in underexposure and the squandering of a significant amount of advertising fees. For instance, the quarter-final match between England and France in the FIFA World Cup 2022 garnered only a viewership of 1.047% in mainland China, which is a disappointing result from an investment standpoint. Isolating predictors in real-world scenarios is very challenging, as evidenced by the dearth of relevant literature on the subject. Small sample populations from Chile (*N* = 2,100) (5) and Swiss (*N* = 4,160) (6) diminish the universality of the conclusions of the very few publications on this subject. In the orphan publication based on the national-level viewership ratings, Kim and Kim analyzed the FIFA World Cup 2006 data and concluded that the participation of national teams and local time of day for a live broadcast are significant predictors of viewership ratings (7). From a strictly statistical standpoint, their conclusions remain debatable. Kim and Kim used least square regression for their analysis and subsequent conclusions. The paper did not explicitly mention whether the response variable (i.e., viewership ratings) conforms to a Gaussian distribution. In our statistical practice, we often observe that real-world data do not always match the underlying assumption. When the statistical assumption is violated, it can result in erroneous conclusions. Viewership ratings are the result of pluralistic consumer psychology-driven behaviors, making forecasting extremely difficult. Thus, a heteroscedastic regression model capable of modeling both the location and the dispersion with explanatory variables seems to be the method of choice.

From a financial perspective, the mainland China market has already emerged as a crucial source of revenue for FIFA. Chinese enterprises committed $835 million towards advertising at the FIFA World Cup 2018, accounting for 35% of FIFA's sponsorship that year and surpassing the $400 million expenditure by American enterprises (8). Chinese enterprises did not hold back during the FIFA World Cup 2022, investing $1.4 billion in advertising and sponsorship (9) to showcase their commercial stance on the event. In light of the enormous financial ramifications of the FIFA World Cup, filling the knowledge vacuum about the driving factors of viewership ratings is of immense theoretical and practical significance.

Furthermore, the course of globalization and its system of governance are currently undergoing a radical transformation in the 2020s. Therefore, it is increasingly crucial to foster peace through sports (10), and soccer stands out as the most suitable choice for this purpose. From the perspective of promoting peace, understanding the influential driving factors and enhancing the viewership ratings of FIFA World Cup 2026 in China holds greater significance than the economic aspects for the Sino-US relationships in this decade.

As aforementioned, modeling the viewership ratings is not straightforward due to complex consumer behaviors, necessitating the use of statistical procedures that are more flexible to real-world data structures. To tackle this research and practical challenge, this study used the generalized additive models for location, scale, and shape (GAMLSS) (11) to identify Chinese consumer behavior during the FIFA World Cup 2022. Despite granting sub-license agreements to six other live broadcasters in China, CCTV's live broadcast of the FIFA World Cup remains dominant. Therefore, this study analyzed the viewership ratings of the FIFA World Cup 2022 on CCTV as the response variable. The objective was to quantify the driving factors and recommend tailored marketing strategies for the FIFA World Cup 2026.

## 2 Methods

### 2.1 Explanatory variables

It is common knowledge that factors such as broadcasting time and day of the week affect viewership ratings. Even more complex and diverse factors exist in sports. For example, a team forms its brand and influences its fan base (12) and, in turn, the viewership ratings. When competing at the national level, a team not only symbolizes its sporting identity but also embodies a cultural identity, such as the Brazilian men's soccer team in the world of soccer. This cultural identity may transcend beyond the team's physical boundaries (13). Hence, it is necessary to include potential covariables in the statistical modeling process and determine individual interpretations for each covariable.

Table 1 outlines the covariables analyzed in this study. Generalization and specificity were taken into account when considering potential driving factors. van Reeth and Osokin (14) proposed that as the major soccer tournaments progress, such as the FIFA World Cup and European Championship, so does TV viewership. Accordingly, matches are coded as either group or knock-out to reflect this dynamic. The schedule plays a crucial role in determining viewing ratings, regardless of whether it is for sports or non-sports TV broadcasts. For this study, weekend matches are specifically defined as matches that take place from Friday evening at 1,800 h through Sunday evening at midnight. TV prime time in China is between 1,900 h and 2,100 h. Matches that began at 1,800 h are also coded as prime time because they occurred inside this timeframe. Of note, the match kick-off time (as Beijing time) is coded as a continuous covariable, as the aim of our modeling is not only to determine if there is a significant prime time effect but also to understand the impact of time dynamics on viewership ratings. Upon examining the literature, evidence from Italian Serie A TV broadcasts suggests that matches between top-ranked teams are often attractive to Italian consumers (15). The hypothesis of this study posited that Chinese consumer demand may be enhanced by both historical and recent triumphs in international soccer competitions. Among these factor-type covariables, it is of interest to determine whether South Korean or Japanese matches exert an influence on viewership ratings. These two Eastern Asian nations are regional soccer powerhouses, and as direct rivals of the Chinese team in the FIFA World Cup and Asia Cup qualifiers, Chinese soccer enthusiasts track their international performance. The FIFA world ranking is a technical point system that is mostly known by devoted soccer lovers. This indicator is chosen to determine whether higher-level matches, specifically those with a higher combined points total between two rival teams, would attract a larger audience. Meanwhile, the point differential between the two rival teams somewhat mirrors the discrepancy in their respective degrees of competitiveness. Consumers may anticipate that a match's outcome will be less predictable if the two sides are closely matched in terms of competitiveness, and conversely. Hence, we calculate the difference in FIFA World Ranking points between the two rival teams as a metric for the level of uncertainty in the match. The soccer star celebrity effect has been observed in both established (15) and emerging (16) soccer lands. In light of this, the team market value (i.e., the combined values of two rival teams) is used to quantify the team-level celebrity effect. In addition, based on a survey of 12,347 Chinese soccer fans conducted in February 2012 (17), the top five favorite international players among Chinese fans were Cristiano Ronaldo from Portugal, Lionel Messi from Argentina, Kylian Mbappé from France, Erling Haaland from Norway, and Robert Lewandowski from Poland. Accordingly, the four teams that the four players represented are coded as popular star teams to examine the individual-level celebrity effect.

**Table 1:** Factors driving the viewership ratings of the FIFA world cup 2022 in mainland China.

Covariable	Type	Range	Source
Tournament stage	Factor	“group”; ‘knock-out”	Open
Weekend	Factor	“No”; “Yes”	Open
Prime time	Factor	“No”; “Yes”	Open
FIFA World Cup champion	Factor	“No”; “Yes”	Open
UEFA Euro Cup champion	Factor	“No”; “Yes”	Open
South Korea or Japan	Factor	“No”; “Yes”	Open
Top 4 in FIFA 2018	Factor	“No”; “Yes”	Open
Popular star in China	Factor	“No”; “Yes”	Open
Match kick-off time	Numeric	03:00 to 24:00, in hrs	Open
FIFA world ranking	Numeric	2,904.28 to 3,533.66, in points	FIFA^a^
Uncertainty of match	Numeric	5.21 to 369.86, in points	FIFA^a^
Team market value	Numeric	6.75 to 2,290.00, in million €	Transfermarkt^b^

^a^ Total points data documented as of October 6, 2022.

^b^ Valuation data accessed as of March, 2023.

### 2.2 Modeling procedures

The present analyses used the gamlss package in the RStudio. The GAMLSS contains more than 100 flexible distributions and is growing. For this study, the viewership ratings range between 0 and 1, and accordingly, the following seven explicit distributions were considered for fitting the response and covariables: beta distribution (BE and BEo), zero-inflated beta (BEINF0, BEINF1, and BEOI), Logit Normal, simplex, and gen beta type 1. Among these, BE, BEo, LOGITNO, and SIMPLEX are two-parameter distributions that model location and scale, whereas the remaining distributions can be used to model the shape parameter(s). Given that two covariables, FIFA world ranking and uncertainty of match, are derived from the same point system, there is a potential concern about collinearity. To tackle this problem, we modified the fitting algorithms to the RS-CG algorithm, which is particularly suited for fitting distribution parameters that exhibit strong correlations. To evaluate the goodness-of-fit of different distributions, we compared the Akaike information criterion (18), the chi-squared (i.e., *k* = 3.84), and the Schwarz Bayesian criterion (19). The model with the smallest values for these criteria was chosen as the marginal distribution. Considering the range and number of observations in this study, we used the default link functions for the moment-based measures.

We adopt a stepwise strategy described by Ramires and colleagues (20). Briefly, a forward selection procedure was applied based on the marginal distribution and assigned link functions to select linear terms for μ, σ, ν, and τ as appropriate, for each distributional parameter. This is then followed by a backward selection procedure for τ, ν, σ, and μ in order, as appropriate. In the modeling, a more restrictive penalty (i.e., *k* = 3.84) was used to select additive terms. Because there are only 64 observations, no attempt was made to smooth continuous covariables. In addition, the two-way interaction model is not taken into account due to its little contribution to explanatory power and the challenge of interpretability associated with complicated statistical modeling. As a final step, the conditional distribution was diagnosed through normality tests, visual inspection of the normalized quantile residuals, and the worm plot. Since one covariable is considered time series data, autocorrelation was also examined.

## 3 Results

### 3.1 Marginal distribution

Table 2 provides a summary of the normality test for viewership ratings based on their fit to selected distributions. The tests validated our hypothesis that the response variable is non-Gaussian. According to the moment-based measures, it has a skewness of 1.610 and a kurtosis of 5.280. The use of a more flexible distribution is warranted.

**Table 2:** Normality test results.

Distribution	*p*-value		
	Shapiro–Wilk	Anderson–Darling	Cramér–von Mises
Normal	0.0005596	0.001079	0.002399
Logit Normal	0.9629	0.9158	0.8659
beta	0.2998	0.3636	0.3895

We started fitting the response variable to alternative distributions, such as beta and Logit Normal proposed by Stasinopoulos and Rigby (21), which both improved the fit of the data (Table 2). Next, we fitted the response variable with a regression structure. Using either criterion, the Logit Normal distribution was deemed “best”. Accordingly, the subsequent analyses were fixed using the Logit Normal distribution.

### 3.2 Conditional distribution

Table 3 presents the estimated linear structure of the fitted model following the stepwise strategy.

**Table 3:** Maximum likelihood estimators of the parameters, SEs, and *p*-values of the logit normal regression.

Parameter	MLE	SE	*p*-value
μ			
Intercept	−4.874	0.099	<0.001
Match kick-off time	0.043	0.006	<0.001
σ			
Intercept	−14.28	0.573	<0.001
FIFA World Cup champion	−1.348	0.379	0.002
FIFA world ranking	0.004	0.000	<0.001
GD = −185.687; AIC = −175.687; SBC = −169.797			

AIC, Akaike information criterion; GD, global deviance; SBC, Schwarz Bayesian Criterion.

The distributional regression based on the Logit Normal distribution is given by (Eq. 1):

(1) $$\begin{array}{c} Y=\mathrm{LOGITNO}(\hat{\mu },\hat{\sigma })\\ \hat{\mu }\;=\mathrm{logistic}[-4.874+0.043\times \mathrm{match}\;\mathrm{kick}\text{-}\mathrm{off}\;\mathrm{time}]\\ \hat{\sigma }=\exp[-14.26\text{–}1.346\;(\mathrm{if},\mathrm{FIFA}\;\mathrm{World}\;\mathrm{Cup}\;\mathrm{champion}{=}^{\prime }{\mathrm{Yes}}^{\prime })\\ +0.004\times \mathrm{FIFA}\;\mathrm{world}\;\mathrm{ranking}] \end{array}$$

**Figure 1:**
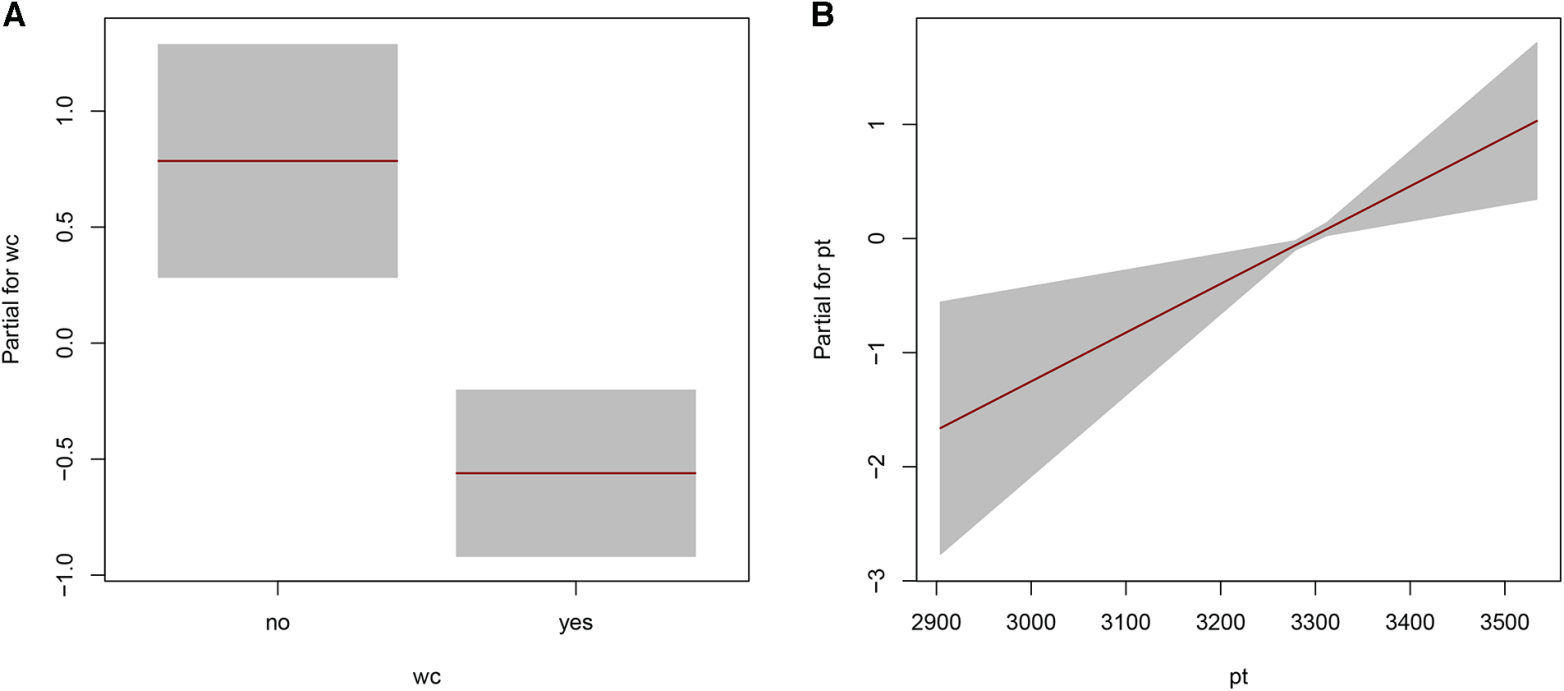
The partial effects of the FIFA world cup champion (**A**) and FIFA world ranking (**B**) on the viewership ratings of the FIFA world cup 2022 in mainland China.

In the model, only one covariable was selected to model the location parameter. Based on the median μ, the viewership ratings of the FIFA World Cup 2022 increased as times of the day. Meanwhile, two covariables were included to model the parameter σ. Figure 1 illustrates that the dispersion decreased for those matches played by FIFA World Cup champions, while an increase in dispersion is observed for teams ranked higher in the FIFA world ranking. In other words, viewership ratings tend to be more stable for matches involving Argentina, Brazil, England, France, Germany, Spain, and Uruguay.

### 3.3 Diagnosis

**Figure 2:**
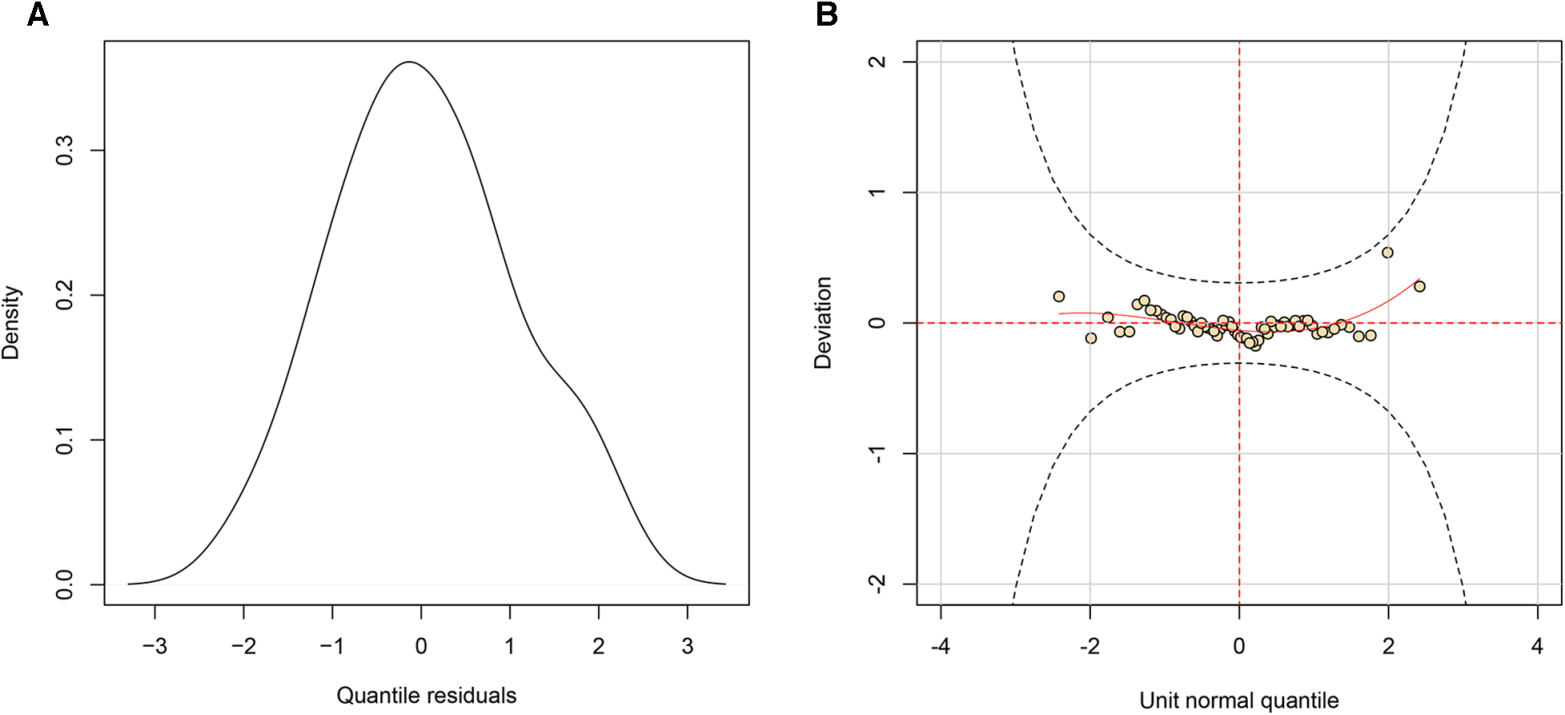
Residual analysis of the viewership ratings of the FIFA world cup 2022 in mainland China. (**A**) the density of the quantile residuals; (**B**) the worm plot of the residuals.

**Figure 3:**
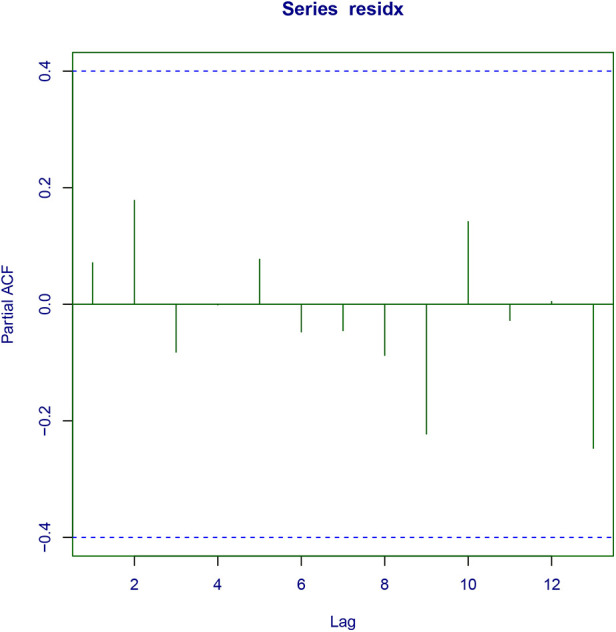
Partial autocorrelation function plot.

As a final step in the modeling process, we assessed whether the residuals of the conditional model support the assumed response distribution. Upon visual examination of the quantile residuals (Figure 2A), it is evident that the quantile residuals have a nearly normal distribution. This is supported by a Filliben correlation coefficient of 0.994. In addition, the worm plot (Figure 2B) shows that all points lie between the two elliptic curves with no particular shape, confirming that the Logit Normal regression model is adequate. Lastly, Figure 3 depicts the partial autocorrelation function. Since all values lie within the confidence intervals, the time series can be concluded to be random.

## 4 Discussion

This study analyzed CCTV viewership ratings that collectively covered more than 2 billion Chinese TV consumers during the FIFA World Cup 2022. We applied distributional regression to model the complex consumer behaviors, with the Logit Normal regression effectively capturing the dynamics of Chinese consumer preference. Our findings are conducive to the realization of time- and team-sensitive marketing strategies for the FIFA World Cup 2026.

### 4.1 Kick-off time matters

There are several hypotheses in the academic literature concerning soccer TV viewership ratings. The so-called uncertainty of outcome was a prevalent theory that has been refuted by several convincing empirical data from mainstream European leagues (22, 23). According to data from the United States (16) and Europe (15), the qualities of match fuel TV viewership ratings, which makes sense in the Western media industry, where on-demand pay-per-view is the predominant business model. Modern sports heavily rely on celebrity influence for branding purposes. It has been revealed that star quality plays a crucial role in driving TV demands in mainstream soccer leagues (15), and this trend is also evident among Chinese soccer fans (24). However, this study found that factors such as the tournament stage, the team's status as the champion of the FIFA World Cup or UEFA Euro Cup, being in the top 4 in FIFA 2018, and the FIFA world ranking, which are often seen as indicators of match quality, did not have an impact on the viewership ratings. Likewise, the team market value, which is determined by the team's star supremacy, does not have any impact. Previous research investigating the impact of match or star quality on viewership ratings focused on soccer fans who have special preferences for TV viewing. In contrast, the modern FIFA endeavors to broaden the sport's popularity beyond just soccer enthusiasts, presenting it as a sustainable lifestyle choice (25). Hence, the audience base is and should be much more diverse in terms of socioeconomic and cultural contexts, which may have nullified any match quality or celebrity effect when sampling a broad population.

Our regression model indicates that the Chinese viewership ratings are driven by the match kick-off time, aligning with the preferences of Korean TV consumers during the FIFA World Cup 2006 (7). In other words, matches played between 18:00 and 24:00 h Beijing time received superior ratings compared to those played after 24:00 h Beijing time. This phenomenon is consistent with the viewing patterns of the broad Chinese TV audience. Chi and colleagues adopted a dynamic panel model to estimate the viewing behavior of Chinese TV consumers and concluded that only the time of day affected viewership ratings (26). Collectively, our finding indicates that the general Chinese population's TV viewing expectations are not influenced by either cultural or domain-specific recognition, such as the FIFA World Cup.

Consequently, our findings indicate that the marketing aspect of the FIFA World Cup 2026 will pose significant challenges for both international organizers and Chinese enterprises. As aforementioned, the mainland China market has become one solid revenue pillar for FIFA. Under President Gianni Infantino's leadership, as outlined in “FIFA 2.0: The Vision for the Future” (27), FIFA is committed to promoting the role of soccer in breaking cultural and social boundaries, therefore ensuring that the sport is accessible to all.

As the world enters the 2020s, a period to date characterized by socioeconomic inequality, ideological and actual conflicts, and de-globalization, these efforts and endeavors are increasingly vital for sustainable global governance. As a result, the popularity of the FIFA World Cup entails a greater diplomatic obligation to foster global harmony (28). From a holistic standpoint, this is in line with the Chinese government's policy of advancing health, common prosperity, and global peace and development through sports (29). FIFA is proactively promoting soccer worldwide, including among China's 1.4 billion citizens, reshaping the FIFA World Cup into a peace-promoting event (30).

Understanding this much broader social background, the kick-off time of the FIFA World Cup 2026 will hold significant importance for FIFA and local organizers in their efforts to promote soccer coverage as a vehicle for global peacemaking. Since the official tournament schedule has not been finalized, this analysis provides the FIFA leadership with a fresh viewpoint to contemplate. During the group stage of the FIFA World Cup 1994, matches began as early as 11:30 EST (Eastern Standard Time) and as late as 19:00 EST, while knock-out matches were played between 9:30 and 13:00 EST. If these kick-off times were replicated for the upcoming FIFA World Cup 2026, our analysis suggests that the viewership ratings for the group stage match (corresponding to 23:30–07:00 Beijing time) would be rather low in mainland China market, whereas the knock-out stage matches (corresponding to 21:30–01:00 Beijing time) would be relatively more convenient for Chinese consumers. Nevertheless, if the local organizers engineer all matches during the local late afternoon and evening hours in North America, it could result in low viewership ratings in mainland China and likely the rest of Asia, where more than half of the world's population resides. This would not align with FIFA's marketing and broader social objectives. The schedule for a global sporting event is improbable to satisfy the preferences of every nation. The authors advocate that in order to promote global health, inclusivity, and peace, North American organizers and consumers share a small amount of convenience by scheduling more matches in the morning to early noon of North American time. This would allow for more international guests to watch the matches on their TV and mobile devices.

Even though this is not the first time mega-events have been held in North America, marketing strategies are constantly evolving in the digital era. Chinese businesses, including both vendors and buyers of advertising, would inevitably face key challenges in managing their business decisions. Advertising buyers, especially those targeting solely the mainland China market, expect a diminished ratio of profit to input and may be less inclined to engage in a bidding “war” for prominent advertisement slots. In the meantime, the Chinese marketing platform should not anticipate a discount from FIFA or its local broadcasting rights holder (i.e., CCTV) and will have a more challenging time marketing those advertisement slots at inconvenient match kick-off times. In the least favorable scenario, where matches are scheduled for the local late afternoon and evening hours in North America, we recommend that Chinese advertising buyers, particularly those aiming to gain access to overseas markets, adopt a long-term strategy and manage the FIFA World Cup sponsorship as a continuing sports asset as opposed to an event-driven traffic advertising for domestic and international markets. On the other hand, advertising vendors may contemplate adopting a differentiated pricing strategy to counterbalance any challenges in selling advertisements. Our analysis suggests that broadcasting matches in the early evening Beijing time could lead to higher viewership ratings, allowing for the possibility of marketing them at a premium price. As the final tournament schedule is yet to be determined, businesses have an opportunity to develop their counter-strategies. This study will help them make evidence-based decisions for Chinese brands, both emerging and established, that are contemplating growth within the country and internationally.

### 4.2 Champions of the world cup with greater investment security

Importantly, the regression model illustrates the presence of two focal points for data variations. On the one hand, matches contested by FIFA World Cup champions exhibit reduced data dispersion; therefore, FIFA World Cup champions have a stabilizing effect on viewership ratings in the mainland China market. Although there is no direct analysis focused on the FIFA World Cup to support the first point, alternative evidence from Spanish La Liga viewership ratings corroborates the current conclusion. Pérez and colleagues analyzed La Liga seasons from 2008 to 2012 and found that, except for El Clásico matches, there was substantial variation in the number of TV viewers (23). Real Madrid and FC Barcelona are not only the dominant La Liga champions but also two of the most well-known soccer clubs in the world. In turn, their matches attract stable viewership, demonstrating a pattern where the winners attract even more spectators, a phenomenon that is also found in other sports (31). The same can be said for the FIFA World Cup champions.

In contrast, trends in the corresponding data point to continued elevated ratings dispersion, despite the overall increase in FIFA world ranking. This suggests that Chinese consumer psychology is not affected by the inclusion of more technical aspects in the FIFA World Cup. To illustrate this argument, we present the results of six matches that were held at 23:00 h Beijing time. The “bottom” three matches (Ghana vs. Uruguay; Canada vs. Morocco; Ecuador vs. Senegal) with the lowest FIFA world ranking (M = 3,040 points) had average viewership ratings of 1.24% (SD = 0.22%), while the “top” three matches (Croatia vs. Belgium; Croatia vs. Brazil; Argentina vs. France; M = 3,494 points) with the highest FIFA world ranking (M = 3,494 points) had average viewership ratings of 3.34% (SD = 2.06%). The implication is double-sided. In one respect, matches contested by lower-ranked teams continue to be less appealing to Chinese TV viewers, resulting in low viewership ratings and suppressed variations among these teams. Nevertheless, if viewership ratings improve with FIFA world ranking, we would expect viewership ratings to be more stable, which is not the case. In other words, when highly ranked teams compete, the variance increases, reflecting the complex consumer psychology underlying team preference. Notwithstanding the FIFA world ranking providing a scientific classification of athletic performance, such a technical perspective does not affect the overall preference of Chinese TV consumers. Possibly, modern sports consumption is driven by Elias and Dunning's theory of sport and excitement (32), and a higher ranking does not necessarily indicate a greater level of enthusiasm among a large, non-professional audience. Taken together, only the FIFA World Cup champions can guarantee the match's interest among Chinese TV viewers, which stabilizes viewership ratings.

Business management constantly strives for certainty and our results are of major practical implications. During the FIFA World Cup 2018, Chinese Vatti became one of the official sponsors of the French team and launched a nationwide advertising campaign stating, “If France wins the championship, Vatti will refund the entire purchase price,” making it the marketing highlight of that World Cup in China. Based on both empirical evidence and our analysis, it is advisable for Chinese enterprises to sponsor the FIFA World Cup champions during live TV broadcasts and offline advertising. Given that those viewership ratings for the FIFA World Cup 2026 will be highly dependent on the kick-off time, our strategy will provide investment security by ensuring certainty during times of elevated uncertainty. This does not imply, however, that non-champion teams are not worth investing in, especially when a business seeks to enter a specific market (33). In the meantime, our findings indicate a unique fluctuation opportunity for advertising vendors to capitalize on those matches contested by FIFA World Cup champions, and our team-sensitive strategy could also be leveraged by those champion teams when negotiating with interested businesses.

### 4.3 Limitation

While this study fills an empty position in the literature, it also has a few limitations that will compromise the efficacy of the proposed strategies. Firstly, the primary “audience” of this study is Chinese enterprises; consequently, all data and conclusions are China-specific and may not apply to consumer behaviors in other regions. With only 64 observations, we did not attempt to fit smoothing additive terms, and the resulting model contains only linear terms of explanatory variables; as a result, this fully parametric model may underfit the data. In the meantime, it should also be acknowledged that the GAMLSS framework allows us to demonstrate reasonable analytic power and present the strategies. The primary drawback may result from the swift evolution of media broadcasting. The present conclusions are founded on the analysis of CCTV broadcasting, which was provided at no cost to Chinese television viewers. However, as more market participants enter live sports broadcasting, such as the short-video, pay-per-view model, consumer behaviors will likely be strongly affected by these changes, which will likely render some of our conclusions and strategies invalid. Chinese enterprises must closely monitor the evolution of media dissemination and adjust their strategies accordingly. Lastly, we caution that regardless of how reliable a model may appear, future business outcomes in mainland China will be profoundly impacted by political uncertainty between China and the United States.

## 5 Conclusion

We show that match kick-off time provides a plausible mechanism for explaining the viewership ratings dynamics of the FIFA World Cup 2022 in mainland China. The regression model further indicates that matches contested by the FIFA World Cup champions served to suppress the dispersion of viewership ratings, whereas an increase in the FIFA world ranking was associated with a rise in dispersion. Understanding the factors driving viewership ratings is of the uttermost importance for optimizing investment decisions in the upcoming FIFA World Cup 2026 for a more certain and superior business return. Ultimately, the proposed strategies are intended for greater FIFA World Cup coverage as a vehicle for peace promotion.

## Data Availability

The raw data supporting the conclusions of this article will be made available by the authors, without undue reservation.
